# Increased NMDA receptor inhibition at an increased Sevoflurane MAC

**DOI:** 10.1186/1471-2253-12-9

**Published:** 2012-06-06

**Authors:** Robert J Brosnan, Roberto Thiesen

**Affiliations:** 1 Department of Surgical and Radiological Sciences, School of Veterinary Medicine, University of California, One Shields Avenue, Davis, CA, 95616, USA

## Abstract

****Background**:**

Sevoflurane potently enhances glycine receptor currents and more modestly decreases NMDA receptor currents, each of which may contribute to immobility. This modest NMDA receptor antagonism by sevoflurane at a minimum alveolar concentration (MAC) could be reciprocally related to large potentiation of other inhibitory ion channels. If so, then reduced glycine receptor potency should increase NMDA receptor antagonism by sevoflurane at MAC.

****Methods**:**

Indwelling lumbar subarachnoid catheters were surgically placed in 14 anesthetized rats. Rats were anesthetized with sevoflurane the next day, and a pre-infusion sevoflurane MAC was measured in duplicate using a tail clamp method. Artificial CSF (aCSF) containing either 0 or 4 mg/mL strychnine was then infused intrathecally at 4 μL/min, and the post-infusion baseline sevoflurane MAC was measured. Finally, aCSF containing strychnine (either 0 or 4 mg/mL) plus 0.4 mg/mL dizocilpine (MK-801) was administered intrathecally at 4 μL/min, and the post-dizocilpine sevoflurane MAC was measured.

****Results**:**

Pre-infusion sevoflurane MAC was 2.26%. Intrathecal aCSF alone did not affect MAC, but intrathecal strychnine significantly increased sevoflurane requirement. Addition of dizocilpine significantly decreased MAC in all rats, but this decrease was two times larger in rats without intrathecal strychnine compared to rats with intrathecal strychnine, a statistically significant (P < 0.005) difference that is consistent with increased NMDA receptor antagonism by sevoflurane in rats receiving strychnine.

****Conclusions**:**

Glycine receptor antagonism increases NMDA receptor antagonism by sevoflurane at MAC. The magnitude of anesthetic effects on a given ion channel may therefore depend on the magnitude of its effects on other receptors that modulate neuronal excitability.

## **Background**

Several ion channels and cell receptors have been identified as possible targets responsible for the mechanism of inhaled anesthetic action; among these are 3-transmembrane (3TM) receptors, such as N-methyl-d-aspartate (NMDA) receptors, and 4-transmembrane (4TM) receptors, such as γ-aminobutyric acid (GABA) receptors and glycine receptors. At a minimum alveolar concentration (MAC), the magnitude of receptor potentiation or inhibition by a given anesthetic agent differs greatly between receptor types [1,2], implying that the respective contributions of these receptors to anesthetic endpoints such as immobility and amnesia likewise differs. Many inhaled agents show an inverse relationship between efficacy at 3TM and 4TM receptors. For example, cyclopropane and benzene cause >50% NMDA receptor inhibition at MAC, but only potentiate GABA_A_ receptors by <50% at this same concentration. In contrast, halothane and isoflurane potentiate GABA receptors by >350% at MAC while inhibiting NMDA receptors by <30% [1,2].

It is unclear precisely how inhaled anesthetics cause general anesthesia [3]. One possible explanation is that the volatile anesthetics act through multiple receptor targets to inhibit excitatory ion channels and potentiate inhibitory ion channels; the combination of these effects within the spinal cord reduces neuronal excitability to the point of causing immobility at anesthetic concentrations equal to or greater than MAC [4]. If anesthesia results from a summation of ion channel effects in critical neurons, then greater anesthetic potency and efficacy in some receptor types may offset the need for greater contributions from other receptors to anesthesia.

Haloethers, such as isoflurane, sevoflurane, and desflurane, potentiate glycine and GABA_A_ receptors much more than they inhibit NMDA receptors at MAC [5-7]. To the extent that both receptor classes are relevant to anesthetic action, we postulate that the haloethers weakly modulate NMDA receptors at MAC for the reason that they more efficaciously and potently modulate other receptor targets such as GABA and glycine receptors. Conversely, a decrease in efficacy or potency at other important receptor targets may necessitate increased contributions from NMDA receptors to reach the same immobilizing anesthetic endpoint. For example, when the GABA_A_ antagonist picrotoxin is administered intrathecally to decrease isoflurane potency and efficacy at the GABA_A_ receptor, isoflurane binds a greater fraction of NMDA receptors at MAC suggesting that there is greater contribution from this receptor to immobility produced by isoflurane [4].

However, would altering potency of a different ether anesthetic via a different molecular mechanism yield a similar increase in NMDA receptor antagonism at MAC? To answer this question, sevoflurane MAC will be measured in rats during intrathecal administration of either aCSF controls or aCSF + strychnine, a glycine receptor antagonist. Because strychnine will increase sevoflurane MAC and since sevoflurane dose-dependently inhibits NMDA receptors, there should be increased NMDA receptor binding at increased sevoflurane MAC. Next, dizocilpine (MK801), an NMDA receptor antagonist, will be added to the intrathecal infusion and subsequently decrease MAC. Since the strychnine rats should have a greater fraction of NMDA receptors already bound and inhibited by sevoflurane, dizocilpine should decrease MAC less in these animals compared to controls. Such a finding would be consistent with an increased role for NMDA receptors in the anesthetic mechanism of action for sevoflurane at an increased MAC.

## **Methods**

Fourteen male Sprague–Dawley rats (Harlan, Livermore, CA) aged 72 ± 3 days and weighing 277 ± 18 g (mean ± SD) were studied. Animals were housed individually with a 12 hour light/dark cycle and with food and water available *ad libitum*. The University of California Davis IACUC approved this research protocol.

### Interthecal Catheter Placement

Sevoflurane (SevoFlo, Abbot Laboratories, Abbot Park, IL) was used to induce anesthesia in rats within an acrylic chamber. Anesthesia was maintained with sevoflurane in oxygen delivered by facemask and a Mapleson E circuit. The technique used for surgical placement of the intrathecal catheter has been described in detail [4]. Briefly, rats were positioned within a stereotaxic frame with cervical ventroflexion, and the head and neck were shaved, aseptically prepared, and draped. The skin from the parietal bone to the distal spinous process of the axis was incised, and blunt dissection was used to divide and retract the epaxial muscles between the midline facial planes. A 0.5 mm incision was made through the exposed dorsal atlantooccipital membrane through which a 32-gauge intrathecal catheter (Part 0046, RecathCo, Allison Park, PA) was advanced to the mid-lumbar vertebral region. The catheter neck was adhered to deep tissue planes with a drop of cyanoacrylate glue, the exposed catheter lumen was plugged with a 0.35 mm diameter stainless steel wire, and muscles and skin were sutured closed in separate layers.

The incision was infiltrated with 0.15 mL of 1% lidocaine, and 0.5 mg flunixin meglumine was administered IM for analgesia. Rats recovered from anesthesia with facemask oxygen, and heating blankets were used to correct hypothermia. Once awake and moving, rats were returned to their cages; all animals were eating or drinking within 1 hour of recovery and no motor deficits were evident in any animal. Rats were allowed an additional 36–48 hour recovery period prior to the sevoflurane MAC studies.

### Intrathecal solution preparation

Animals were assigned to either a control group or the strychnine group, and each experiment was conducted in the following sequence: Baseline MAC measurement (no intrathecal solution administered), Pre-dizocilpine MAC measurement (strychnine or control intrathecal solution administered), and Dizocilpine MAC measurement (strychnine + dizocilpine or control + dizocilpine).

Study solutions were prepared daily using artificial cerebrospinal fluid (aCSF) perfusion fluid (Harvard Apparatus, Holliston, MA) and sterile syringes. For the Pre-dizocilpine measurements, a solution of 4 mg/mL L-strychnine (98%, Acros Organics, Geel, Belgium) was prepared in aCSF within a 10 mL additive-free, stoppered blood collection tube. In order to solubilize strychnine at this concentration, a 10 ml syringe and needle were used to create a vacuum within the tube, and the headspace was replaced with 99.99% CO_2_ (Matheson, Newark, CA). This process was repeated for a total of 10 headspace exchanges and resulted in approximately 1 atm CO_2_ tension. The control pre-dizocilpine solution containing aCSF alone was similarly acidified with CO_2_ headspace exchanges and measured using the same gas analyzer.

The solutions for the dizocilpine measurements were prepared under sterile conditions in a manner identical to that described for the pre-dizocilpine solutions, except for the addition of 0.4 mg/mL (+)-MK801 hydrogen maleate (dizocilpine, Sigma, St. Louis, MO) in both the strychnine and control solutions. Finally, all dizocilpine solutions were acidified with CO_2_ to 1 atm pressure.

### MAC measurement

Rats were individually anesthetized with sevoflurane/O_2_ in 8 × 30 cm acrylic cylinders that were connected in parallel to the fresh gas supply. The inlet was sealed using a one-holed rubber stopper for delivery of oxygen and sevoflurane at a rate of ≥1 L/min per cylinder; a luer adapter allowed a short, stopcock-sealed polypropylene coaxial tube to be used for chamber gas sampling near the nose of the anesthetized rat. The outlet of each cylinder was sealed using a two- holed stopper; rat tails were inserted through one hole until occluded, and a passive waste gas scavenging system was attached to the second hole. A thermistor probe (400 series, YSI, Yellow Springs, OH) that was calibrated against a certified mercury thermometer (SRM934-FC, ERTCO, Dubuque, IA) also traversed a sealed portion of the distal stopcock in order to measure rectal temperature which was maintained between 37 and 38°C using heating pads as needed.

Once each rat was immobilized in its chamber and instrumented with a temperature probe, 3 mL of 0.9% saline was administered subcutaneously to each rat. The delivered sevoflurane concentration was then changed to 2.2-2.5% and maintained constant for 20 min. After equilibration, an alligator clamp was applied to the distal tail and rotated back and forth for 60 sec. The chamber gas was then sampled for analysis by gas chromatography, and the presence or absence of movement in response to tail clamp was recorded. If movement was observed, the sevoflurane concentration was increased by 10-15%. If no movement was observed, the sevoflurane concentration was decreased by 10-15%. After 20 min equilibration at the new sevoflurane concentration, movement in response to tail clamping was re-assessed at a site immediately proximal to the previous test, cylinder gas was sampled for analysis, and the sevoflurane concentration was adjusted up-or-down accordingly. MAC was defined as the arithmetic mean between the highest sevoflurane concentration that permitted movement and the proximate lowest sevoflurane concentration the prevented movement in response to tail clamping. Baseline sevoflurane MAC for each rat equaled the mean of duplicate MAC measurements prior to intrathecal infusions.

Syringes containing pre-dizocilpine solution (either control or strychnine, Table 1) were used to prime extensions sets and blunt needles which were connected to the intrathecal catheters, and the Pre-dizocilpine solution was administered at 4 μL/min via a syringe pump (PHD2000, Harvard Apparatus, Holliston, MA). A 3 mL volume of 0.9% NaCL was also again administered subcutaneously to replace insensible and urinary fluid losses. After a 60 min equilibration time with constant pre-dizocilpine infusion and anesthetic dose, the sevoflurane MAC was re-measured in duplicate using the same tail clamp bracketing technique described for the baseline sevoflurane MAC measurements. All strychnine infusions caused spontaneous and intermittent muscle fasciculation and myoclonus even during apparently deep planes of sevoflurane anesthesia; however only movement that occurred in response to noxious stimulation was considered a positive response endpoint for a MAC bracket [8].

**Table 1:** Intathecal drugs administered to “control” and “strychnine” rat treatment groups during baseline, pre-dizocilpine, and dizocilpine MAC measurement periods

**Group**	**Baseline**	**Pre-Dizocilpine**	**Dizocilpine**
**Control**	no infusion	aCSF+CO_2_	aCSF +CO_2_ + dizocilpine
**Strychnine**	no infusion	aCSF+CO_2_ + strychnine	aCSF + CO_2_ + strychnine +dizocilpine

Next, syringes with primed fluid lines and needles containing the dizocilpine solution (either control or strychnine) were connected to the intrathecal catheters. The dizocilpine solution was again administered at 4 μL/min, and an additional 3 mL of 0.9% saline was administered subcutaneously. After 60 min requilibration time with the dizocilpine infusion and new sevoflurane dose, MAC was re-measured in duplicate using the same bracketing tail clamp technique for the baseline and pre-dizocilpine MAC determinations. All rats were euthanized at the end of the study. The mean total anesthetic time for each experiment was 7.2 hours.

### Gas concentration measurement

Chamber gas concentrations were monitored continuously using a multi-agent infrared anesthetic gas and paramagnetic oxygen analyzer (Andros Model 4800, LumiSense, Orange, CA). For MAC measurements, cylinder gas samples were collected in a glass syringe and analyzed using a gas chromatograph (Clarus 500, Perkin Elmer, Waltham, MA) with direct injection by a 0.25 mL sample loop onto a 183 cm long and 0.32 cm diameter packed SF-96 column at 100°C, a flame ionization detector at 150°C, and ultra-zero air, H_2_ and He flow rates of 350, 35, and 20 mL/min, respectively. The resulting sevoflurane peak had a retention time of 1.23 min, and the area under the curve was integrated using commercial software (TotalChrom, Perkin Elmer, Waltham, MA). The chromatograph was calibrated using secondary sevoflurane gas standards having concentrations previously determined by calibration against multiple primary sevoflurane gas standards that were prepared using the Ideal Gas Law.

### Statistical analysis

Data were described as mean ± SD, and normal distributions of data were verified by visual inspection of normal probability plots and by Shapiro-Wilk tests. Planned contrasts between control and strychnine groups among baseline, pre-dizocilpine, and dizocilpine treatments were analyzed using Student t-tests with Dunn-Sidak corrections for multiple comparisons (v.11, SPSS, Chicago, IL). A P < 0.05 indicated statistical significance.

## **Results**

Sevoflurane MAC in rats prior to intrathecal infusions was 2.26 ± 0.21%atm (mean ± SD). Animals were anesthetized and studied for an average of 7.2 ± 1.8 hours, and duplicate MAC measurements were similar within treatments. The percent change from the first to second sevoflurane MAC measurements, expressed as $100*\frac{MAC_{2}-MAC_{2}}{MAC_{1}}$ for control and strychnine treatments, respectively, was: 0.3 ± 0.4 and 0.5 ± 1.6% before aCSF infusions, 1.2 ± 1.1 and 5.1 ± 17.7% during the aCSF infusions without dizocilpine, and −5.5 ± 18.5 and 0.2 ± 3.8% during the aCSF infusions with dizocilpine.

**Figure 1:**
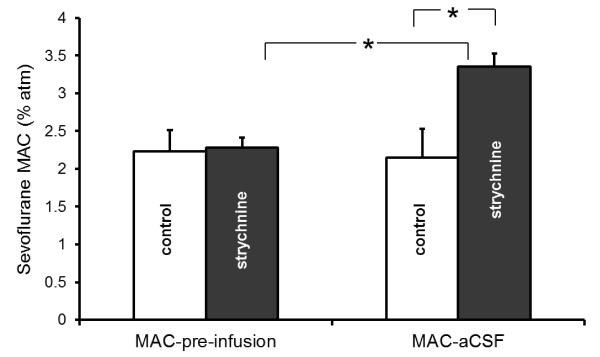
**Sevoflurane MAC before and after intrathecal administration of CO**_**2**_-acidified aCSF either without (control) or with 4 mg/mL strychnine. Statistically significant differences are indicated by an asterisk (*). MAC was unchanged in control animals, indicating that neither intrathecal administration of aCSF nor CO_2_ significantly altered sevoflurane requirement. In contrast, intrathecal strychnine significantly increased sevoflurane MAC.

**Figure 2:**
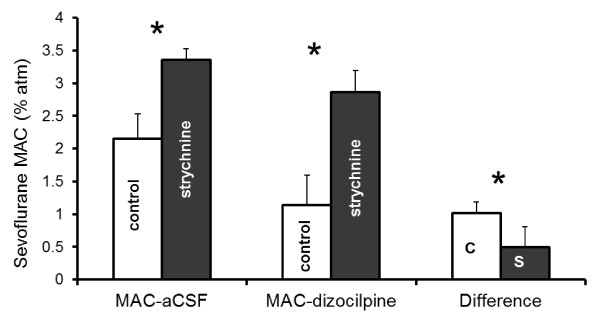
**Sevoflurane MAC during intrathecal administration of CO**_**2**_**-acidified aCSF (MAC-aCSF) and during administration of acidified aCSF containing 0.4 mg/mL dizocilpine (MAC-dizocilpine), either without (control) or with 4 mg/mL strychnine.** The strychnine MAC values were both significantly greater than their corresponding control sevoflurane MAC values (* P < 0.05) The difference in sevoflurane MAC before and after dizocilpine administration is shown in the far right bars for both control (C) and strychnine (S) rats.

Intrathecal strychnine increased sevoflurane MAC by 56% compared to control rats receiving only acidified aCSF (Figure 1). Administration of acidified aCSF alone had essentially no effect on anesthetic requirement; the control sevoflurane MAC during aCSF-infusion was unchanged from the control sevoflurane MAC pre-infusion (Figure 1).Within 10 minutes of administration, strychnine produced intermittent myoclonus in all animals unrelated to noxious stimulation. However, seizure-like activity appeared to decrease or cease following addition of the NMDA antagonist dizocilpine (MK801) to the intrathecal strychnine infusion. Dizocilpine also decreased sevoflurane MAC in both the control and strychnine treatment groups, though MAC in the strychnine group remained significantly greater than for the control group (Figure 2). However, the magnitude of the dizocilpine MAC-sparing effect was significantly different between groups. In the control group, dizocilpine reduced the mean sevoflurane MAC from 2.15 ± 0.38%atm to 1.14 ± 0.46%atm, a change of 1.01 ± 0.17%atm sevoflurane. In the strychnine group, dizocilpine reduced the mean sevoflurane MAC from 3.36 ± 0.17%atm to 2.85 ± 0.33%atm, a difference of only 0.49 ± 0.31%atm sevoflurane. Hence, when sevoflurane MAC was increased using strychnine, the dizocilpine MAC-sparing effect was halved.

## **Discussion**

Intrathecal strychnine increased sevoflurane MAC by 56%. Sevoflurane, like other haloether anesthetics, causes concentration-dependent NMDA receptor inhibition, and the MAC change observed in the present *in vivo* study corresponds to a 10-15% increase in NMDA receptor inhibition based on *in vitro* electrophysiologic studies [9]. Greater NMDA receptor inhibition at increased sevoflurane MAC reduces the population of NMDA receptors available for binding and modulation by other NMDA receptor antagonists such as dizocilpine [7]. As a result, dizocilpine efficacy should be diminished during administration of high sevoflurane concentrations. In fact, this is what we observed in the present study, and it is consistent with increased sevoflurane NMDA receptor antagonism in rats when sevoflurane MAC is increased as a result of glycine receptor antagonism.

Sevoflurane and other ether anesthetics exhibit greater glycine receptor potentiation [5,6] than NMDA receptor inhibition [7,9] at an anesthetic EC_50_ or MAC. However, this does not generally appear to be a fixed property of the ether anesthetics. If inhaled anesthetic immobility is a summation of systemic effects produced by negative modulation of one or more excitatory cell receptors and positive modulation by one or more inhibitory cell receptors [3], then high efficacy at one relevant higher affinity receptor site might render unnecessary the contributions from other potentially relevant lower-affinity sites. The ether anesthetics at MAC may exhibit lower efficacy for some 3-transmembrane receptors, such as NMDA receptors, because immobility may arise primarily through actions at 4-transmembrane receptors, such as GABA_A_ and glycine receptors [10], among other putatively relevant targets [3]. A decrease in haloether potency at 4-transmembrane sites—whether by administration of glycine antagonists during sevoflurane anesthesia as reported here or by administration of a GABA_A_ antagonist during isoflurane anesthesia as described in an analogous study [4] appears to augment the contribution of NMDA receptors (and presumably other relevant spinal targets) to mobility during general anesthesia.

The spinal cord is the primary site for the immobilizing action of inhaled anesthetics [11]; hence receptor antagonists in this study were administered intrathecally rather than systemically. Although exhibiting a ceiling effect [12], strychnine infusions at lower concentrations around the spinal cord in rats dose-dependently increase MAC in a manner that correlates with *in vitro* receptor effects which suggests glycine receptors may contribute to anesthetic immobility [13]. Sevoflurane dose-dependently decreases NMDA-dependent depolarization of nociceptive and non-nociceptive neurons within the rat spinal cord [14] Likewise, dizocilpine dose-dependently decreases MAC in rats to a greater magnitude during spinal infusions compared to either intracerebroventricular or intravenous infusions [15], suggesting that NMDA antagonists are also relevant to the mechanism of action of inhaled agents. Increased NMDA receptor antagonism observed at increased sevoflurane MAC in the present study would be consistent with increased contribution of this receptor to immobility.

Inferences regarding sevoflurane cellular actions have been made with the assumption that pharmacologic manipulation of glycine and NMDA receptor function occur independently of one another. Glycine does cause positive allosteric modulation of NMDA receptors [16], but these glycine binding sites are strychnine-insensitive and therefore should not have been affected during intrathecal strychnine infusions intended to increase sevoflurane MAC. Morerover, neither glycine nor GABA_A_ receptor antagonists affect depression of inward glutamate currents within the spinal cord caused by 1 MAC enfluran [17], and presumably by other ether anesthetics. Similarly, we are unaware of evidence for enhanced spinal endogenous NMDA receptor inhibition caused by spinal strychnine-sensitive glycine receptor antagonism to controvert this assumption of independence.

Sevoflurane MAC measured prior to intrathecal infusions falls within the lower end of published values for Sprague–Dawley rats [18,19]. Intrathecal strychnine prior to dizocilpine administration increased MAC of sevoflurane by 56%, but strychnine infusions maximally increase isoflurane MAC by only 43% [12]. Agent differences are not explained by either receptor affinity or efficacy for the glycine receptor, since the aqueous EC_50_ is lower and the effect maximum is higher with isoflurane versus sevoflurane for their respective logarithm dose–response curves [6]. In contrast, dizocilpine in sevoflurane-anesthetized control rats from the present study produced quantitatively similar MAC-sparing effects when administered intrathecally to isoflurane-anesthetized rats [15].

Strychnine is only sparingly soluble in water at physiologic pH and room temperature [20]. As a Lewis base, strychnine solubility was increased through aCSF acidification with CO_2_. Yet this posed another potential problem. Carbon dioxide is itself an anesthetic in rats, and it dose-dependently decreases MAC of other anesthetics [21]. Furthermore, CO_2_ negatively modulates NMDA receptors [22] via noncompetitive and mixed inhibition at the glutamate and glycine binding sites, respectively [23]. But unlike hydronium ions, CO_2_ moves readily across cell membranes, and a high local transdural partial pressure gradient facilitates CO_2_ diffusion from the CSF to the blood from which it is eliminated via alveolar minute ventilation. This would explain why sevoflurane MAC in control rats receiving intrathecal CO_2_-acidified aCSF was unchanged from the pre-infusion measurements in the same animals (Figure 1). It also suggests that CO_2_ in these experiments was likely not a confounding factor with respect to NMDA receptor function.

Nevertheless, there were important limitations to our study design. Although a control group was used for comparisons, the investigators were not blinded to treatment (aCSF with versus without strychnine) which potentially could have introduced bias in MAC measurements [24]. In initial experiments, attempts were made to blind investigators to the treatment group, but these were ultimately unsuccessful due to myoclonus and an obvious increase in sevoflurane requirement observed in animals that received intrathecal strychnine. In addition, though dizocilpine [25] and strychnine [26] potently inhibit NMDA and glycine receptors, respectively, their effects are not entirely limited to these receptors. Both agents, for example, can modulate acetylcholine receptors [27,28]. To the extent that these or other non-specific receptor interactions contribute to immobility [29], the MAC reduction attributed to NMDA receptor inhibition could be overestimated. The non-NMDA dizocilpine spinal effects, if present in this study, should be similar between control and strychnine rats; therefore the underlying conclusion that loss of glycine receptor effects increases reliance on different anesthetic-sensitive targets to achieve immobility at an increased sevoflurane MAC remains valid.

Immobility may be produced by either anesthesia or toxicity. Generally, anesthetic effects are reversible once the drug is eliminated from the effect site, whereas toxic effects are not. Both strychnine and dizocilpine can be neurotoxic. Reversibility has been previously tested by measuring baseline isoflurane MAC in rats, separately administering strychnine or dizocilpine intrathecally, and then recovering the rats and re-measuring baseline isoflurane MAC two days later. At concentrations equal to or higher than those used in the present study, strychnine and dizocilpine MAC-sparing effects were completely reversible [13,15]. Two other findings suggest that our observations are not a result of toxicity. First, variability between duplicate MAC measurements made for each group and treatment was small. Had toxicity occurred, then the second MAC measurement instead should have been consistently and significantly lower than the first due to progression of spinal injury from continuous intrathecal drug administration. Second, dizocilpine had a smaller sevoflurane MAC-sparing effect in rats receiving strychnine compared to control animals (Figure 2). Had the combination of strychnine and dizocilpine injured the spinal cord, then the exact opposite result would have been expected.

At MAC, the magnitude of NMDA receptor modulation by sevoflurane is increased when contributions of other putatively relevant anesthetic targets to immobility, such as glycine receptors, are diminished. Mutability of receptor contributions to prevent movement during anesthesia is analogous to mutability of neuroanatomic sites of action responsible for this same endpoint. Even though immobility by volatile anesthetics is largely a spinal phenomenon, general anesthesia can nonetheless be achieved when isoflurane is preferentially delivered to the brain rather than the spinal cord, albeit at more than twice the MAC as when the same agent is administered systemically by inhalation [11]. Although isoflurane has the capacity to produce anesthesia via supraspinal sites, it does not for the reason that isoflurane can act at spinal sites with high efficacy and greater potency to cause immobility. Likewise, sevoflurane and other ether anesthetics may be less efficacious NMDA receptor antagonists at MAC for the very reason that they more potently modulate other ion channels and receptors responsible for immobility during anesthesia.

## **Conclusion**

Findings from this study suggest that the magnitude of NMDA contributions to sevoflurane anesthesia may therefore be determined indirectly by sevoflurane efficacy and potency at other relevant cellular targets.

## **Competing interests**

The authors declare that they have no competing interests.

## **Authors’ contributions**

RB conceived and designed the study, helped conduct all experiments, analyzed the data, and wrote the manuscript draft. RT helped conduct all experiments and revised the manuscript draft. Both authors approved the final manuscript.

## **Funding**

This project was funded by a grant from the National Institutes of Health (GM092821-02).

## Pre-publication history

The pre-publication history for this paper can be accessed here:

http://www.biomedcentral.com/1471-2253/12/9/prepub
